# On the impact of Vertical Alignment of MoS_2_ for Efficient Lithium Storage

**DOI:** 10.1038/s41598-017-03453-x

**Published:** 2017-06-12

**Authors:** Victor Shokhen, Yana Miroshnikov, Gregory Gershinsky, Noam Gotlib, Chen Stern, Doron Naveh, David Zitoun

**Affiliations:** 1 Department of Chemistry, Bar Ilan Institute of Nanotechnology and Advanced Materials (BINA), Bar Ilan University, Ramat Gan, 5290002 Israel; 2 Faculty of Engineering, Bar Ilan Institute of Nanotechnology and Advanced Materials (BINA), Bar Ilan University, Ramat Gan, 5290002 Israel

## Abstract

Herein, we report energy storage devices, which are based on densely packed, vertically aligned MoS_2_ (VA-MoS_2_) or planar oriented MoS_2_ (PO-MoS_2_) and compare their electrochemical performances. The VA-MoS_2_ films have been processed by chemical vapor deposition (CVD) to reach unprecedented micron-scale thick films while maintaining the vertical alignment for the whole thickness. The VA-MoS_2_ and the PO-MoS_2_ films form a high-performance Li-ion electrode, reaching the theoretical limits of reversible capacity for this material (800 mAh/g; twice the specific capacity of graphite). The vertical alignment allows faster charge-discharge rates while maintaining a high specific capacity (C-rate measurements). Noteworthy, the reversible cycling of the Li-ion electrode also benefits from the vertical alignment. In this article, we present the full synthesis, structural and electrochemical characterization of VA-MoS_2_ along with the properties of PO-MoS_2_ to deconvolute the intrinsic properties of MoS_2_ from the influence of the layers’ orientation.

## Introduction

Materials such as graphite and layered transition-metal disulfides (LTMDs) are structured from 2D sheets, stacked together by Van der Waals interactions. These rather weak interactions enable the exfoliation of stable two-dimensional sheets by mechanical methods^1–3^ chemical methods^4, 5^ and ultrasonic agitation processing in solution^6, 7^. Apart from the exfoliation methods, growth of ultrathin layered materials by chemical vapor deposition (CVD) has been successfully demonstrated on graphene^8, 9^, hexagonal boron nitride h-BN^10^, molybdenum disulfide (MoS_2_)^11^, tungsten diselenide (WSe_2_)^12^ and more^13, 14^. CVD has been established as an efficient method for the preparation of large crystals with high quality and controllable thickness^15, 16^, together with ALD^17^. In most cases, CVD growth yields 2D materials with basal planes oriented parallel to the substrate, with planar orientation (PO)^18^. This orientation is preferred for the study of the electronic properties of the layers and for the implementation of electronic devices^19^. In particular, the majority of the extensive studies on MoS_2_ growth strive to achieve continuous layers and heterostructures with PO suitable for electronic and optoelectronic devices^20–22^. Layered materials, MoS_2_ in particular, have also been demonstrated as efficient and promising for energy storage applications^23–25^, where the weak interlayer force can accommodate intercalated ions very efficiently. However, for energy storage, the most efficient orientation of MoS_2_ crystals is the vertical alignment (VA) to the substrate, and the desired thickness of the grown layer must be sufficient to insert as many ions as possible, intercalated between the MoS_2_ crystal planes.

CVD has previously led to very thin films of VA-MoS_2_ (5–25 nm) by sulfurization of thin Mo films^26–28^. The transition from PO to VA growth has been rationalized through the investigation of the Mo layer thickness. Above a certain Mo thickness (3 nm), the preferential growth in vertically aligned has been reported for no more than 25 nm thick MoS_2_^29^. VA-MoS_2_ has shown stable electrocatalytic activity for the hydrogen evolution reaction (HER)^28, 30, 31^. However, the intriguing question of its performance as an ion-storing device remains unanswered.

The interaction of alkaline cations in MoS_2_ has been studied from several perspectives, including several device geometries and electrode designs, as well as the choice of cations to be hosted within the electrode. Li-ion batteries are the most used energy source for devices^32–35^. The lithium storage in LTMDs was found to follow a mechanism similar to that of graphite^36^, but with higher specific capacity^23^ at a higher and safer potential^37–39^. The relation of the material geometry and dimensionality to its lithium storage capacity has been extensively studied^40–43^. Exfoliated materials, either graphene or LTMD monolayers^44, 45^, demonstrate very high capacity although we do not fully understand their electrochemical mechanisms^46^. Interestingly, the same processes of intercalation can be used for the preparation of exfoliated LTMDs^23^. MoS_2_ flakes have been tested as Li-ion electrodes with the reversible capacity of 200 mA/g when cycled in the electrochemical window of 1–3 V vs. Li/Li^+^^47^, which matches the intercalation of one Li atom per formula^48^. Such an intercalation can theoretically occur for most of the cations^49^. In contrast, MoS_2_ nanoflakes cycled in a wider potential window of 0.01–3 V show a high reversible capacity of 840 mA/g, corresponding to more than 5 Li/MoS_2_^50^, which does not match the intercalation mechanism, but a full conversion reaction to Li_2_S and Mo^51^. Furthermore, nanocomposite MoS_2_ or graphene show a high discrepancy between the reported reversible capacities in the range of 650–1,500 mAh/g in the electrochemical window of 0–3 V^39, 52, 53^. More generally, the literature does not provide a fully consistent report on the properties of MoS_2_ by itself and on the influence of the crystal orientation on the lithiation properties.

Here, for the first time, we study the performance of Li-storage devices comprised of VA-MoS_2_ and compare the measured capacitance and energy density to those achieved with PO-MoS_2_. For the synthesis of VA-MoS_2_, we use a simple Mo foil compatible with the lithium storage. The sulfurization of Mo into a highly ordered thick layer of VA-MoS_2_ was achieved by a long reaction time. The comparison to PO-MoS_2_ was conducted on films of similar thickness in order to isolate the dependence on crystal orientation from the influence of film thickness on the overall performance of the electrodes.

## Results

### Synthesis

**Figure 1:**
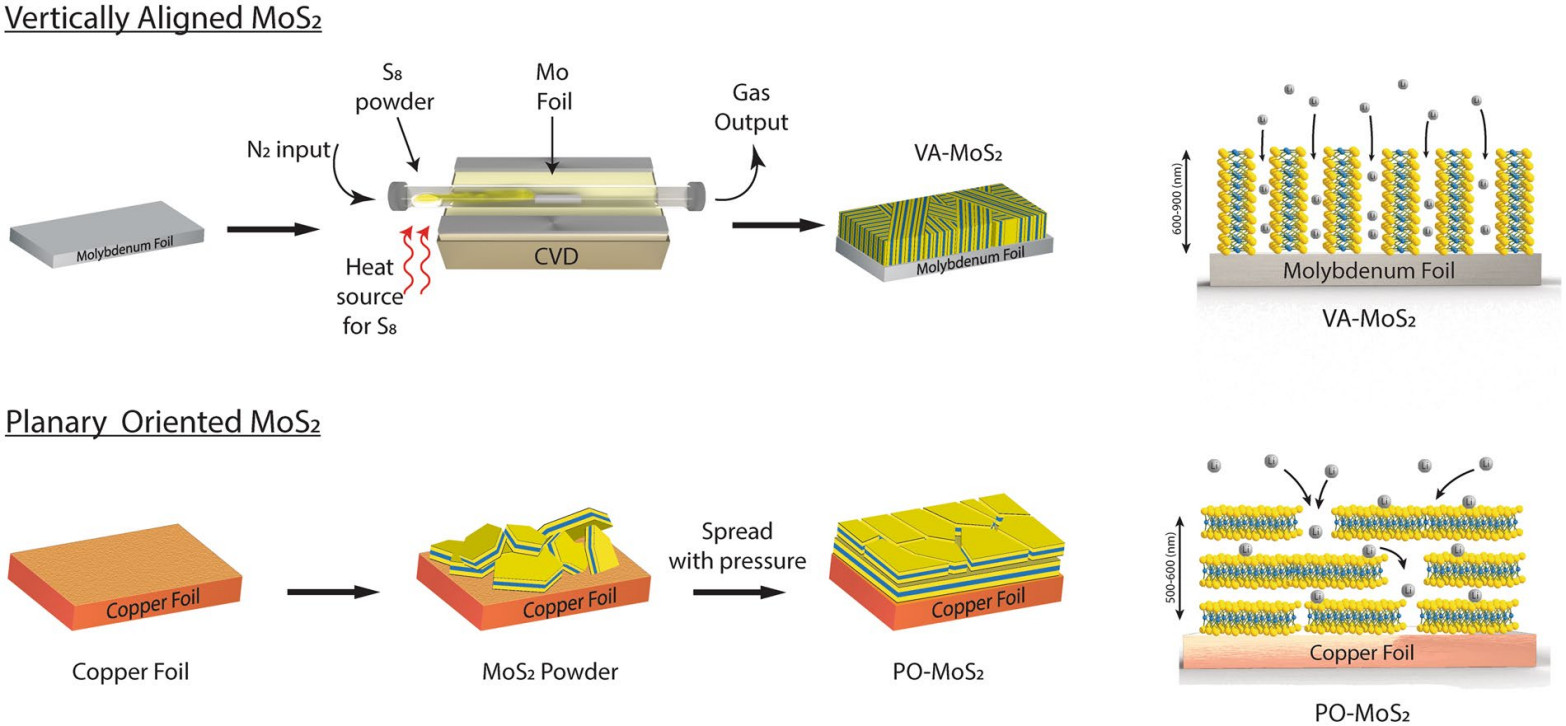
Scheme of the chemical vapor deposition (CVD) experimental set-up and the VA-MoS_2_ electrodes, scheme of the preparation of PO-MoS_2_ electrodes and expected lithiation pathways.

The synthesis of the VA-MoS_2_ was carried out by placing polished molybdenum foil in the CVD furnace (Fig. 1). The sample was purged by nitrogen gas in a thermal annealing step followed by the growth of a MoS_2_ film. During the process, sulfur vapor was introduced into the furnace from a boat filled with sulfur. The reaction was carried out for different durations, leading to several film thicknesses. Henceforth, we refer to the samples of thicknesses 900 nm, 800 nm and 560 nm as VA-MoS_2_ #1, VA-MoS_2_ #2 and VA-MoS_2_ #3, respectively. After the reaction, the foil changed color from silver to dark purple (Fig. S1 in the Supplementary Information). The film thickness was measured by atomic profile analysis through Rutherford backscattering spectroscopy (RBS) (Fig. S2).

### Structural characterization

**Figure 2:**
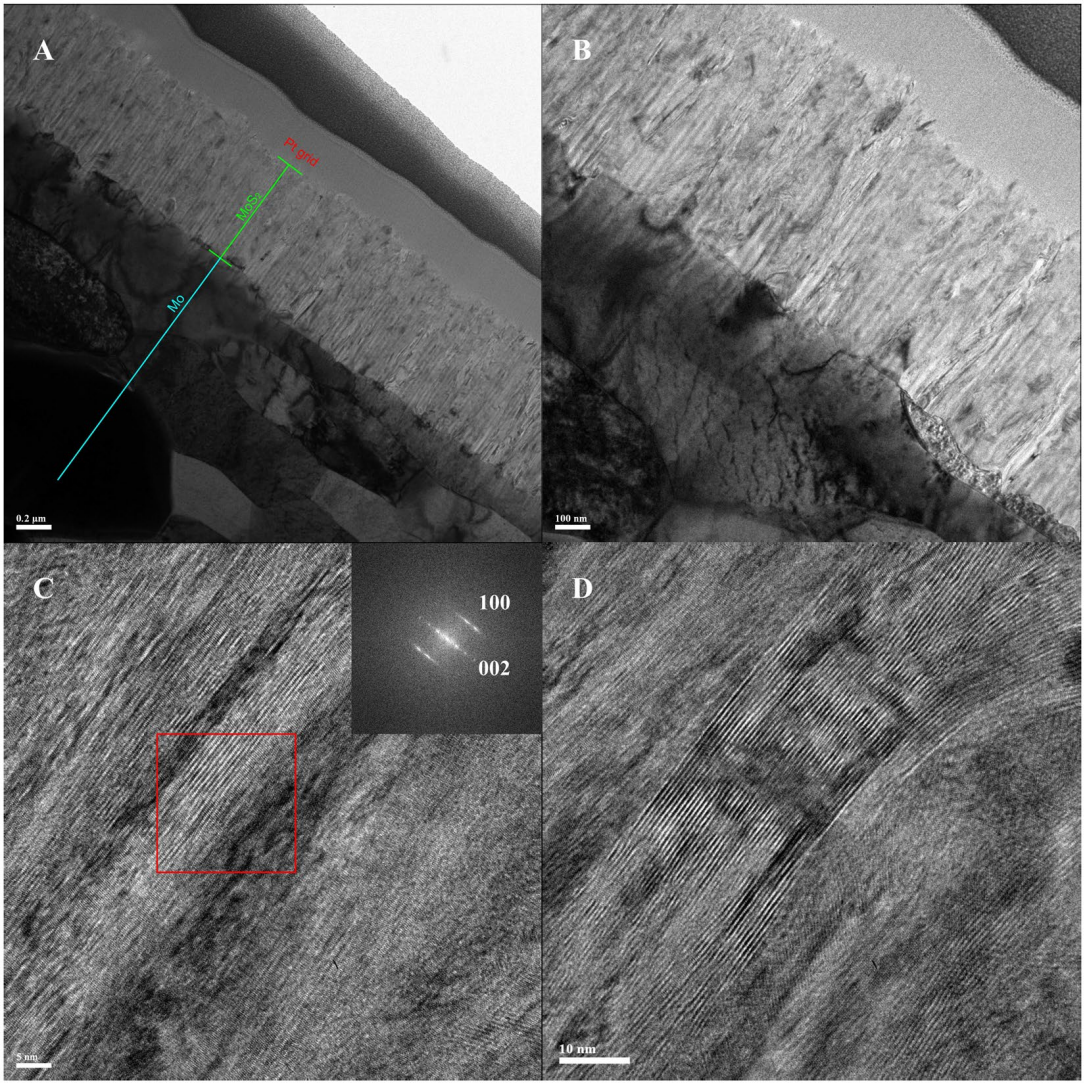
Transmission electron microscopy (TEM) images of VA-MoS_2_ #1 grown on Mo foil with different magnifications (**A**,**B**); high resolution TEM (HRTEM) of several areas and FFT from the selected red square (**C**,**D** and inset).

A slice of VA-MoS_2_ #1 was cut by a focused ion beam (FIB) and the cross-section was imaged and analyzed by high resolution transmission electron microscopy (HRTEM) in a bright field. (Figure 2A), The HRTEM shows the polycrystalline nature of the Mo foil marks as a light blue line and the green line marks the MoS_2_ grown from the surface of the Mo foil and a layer of Pt (added during the FIB process for the purpose of the experiment). VA-MoS_2_ #1 displays a homogeneous thickness of 900 nm throughout the sample.

**Figure 3:**
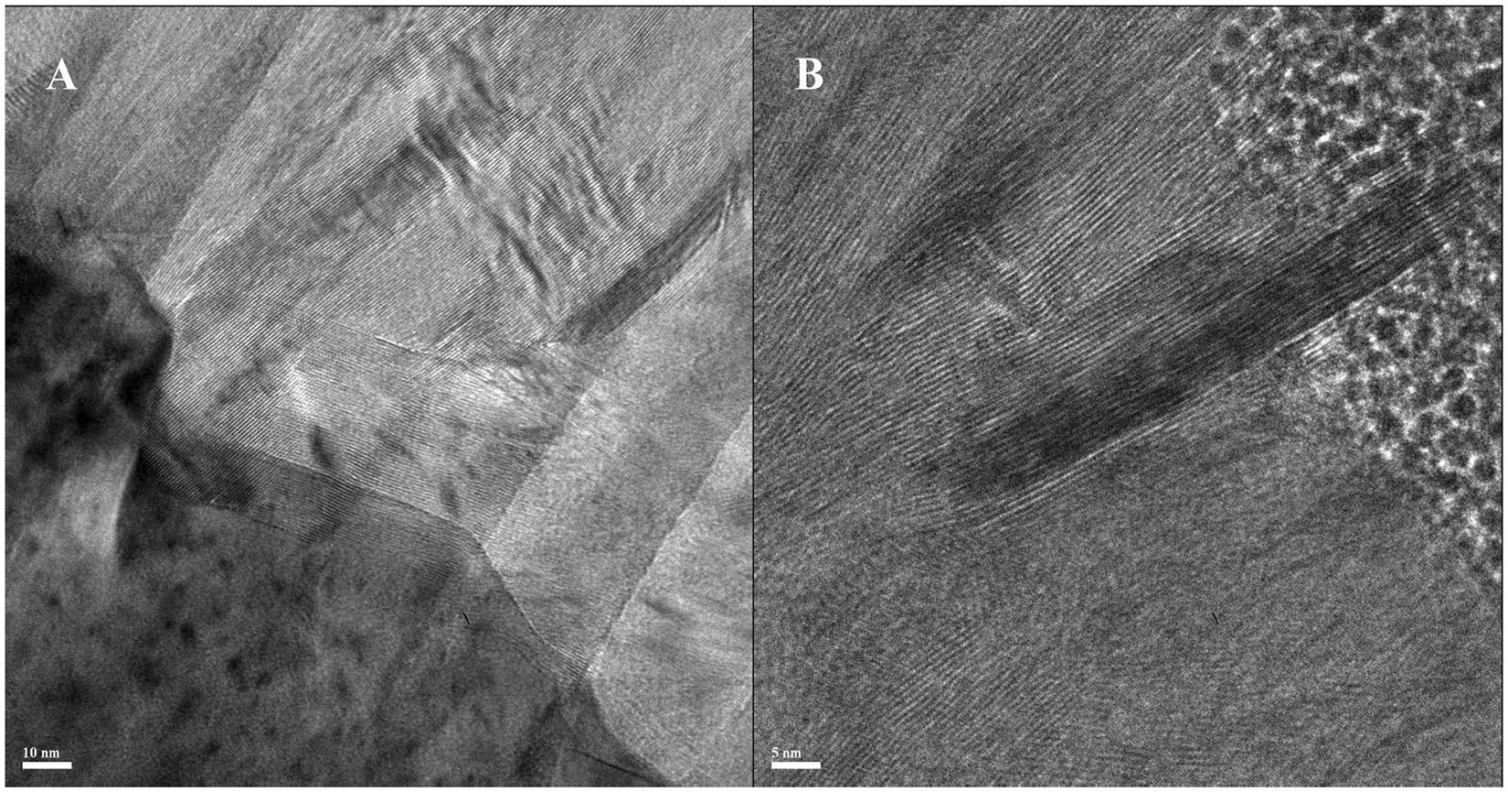
HRTEM of the bottom of the VA-MoS_2_ #1 at the interface with Mo (**A**) and HRTEM of the top of the film (**B**).

Interestingly, the MoS_2_ film displays vertically aligned grain boundaries (Fig. 2B), which arise from the vertical orientation of the film grown from the surface of the Mo foil (Fig. 2A and B). The HRTEM analysis and fast-Fourier transform (FFT) image (Fig. 2C and inset) show lattice fringes of 6.3 Å, corresponding to the (002) basal planes of the 2H-MoS_2_ phase, confirming the vertical alignment of the film. The basal planes were found to be orthogonal to the substrates on all the HRTEM images, showing growth along the (100) plane parallel to the substrate. The almost-perfect VA of the MoS_2_ stabs from the top to the bottom can be clearly observed (Fig. 2D), while a close view shows the abrupt interface between the Mo foil and the MoS_2_ film (Fig. 3A). In some cases, MoS_2_ basal planes grow parallel to the Mo foil close to the interface while, in general the vertical growth starts directly from the interface. On the surface of the film, the layered MoS_2_ opens in a flower-like morphology with a roughness on the order of several nanometers (Fig. 3B).

**Figure 4:**
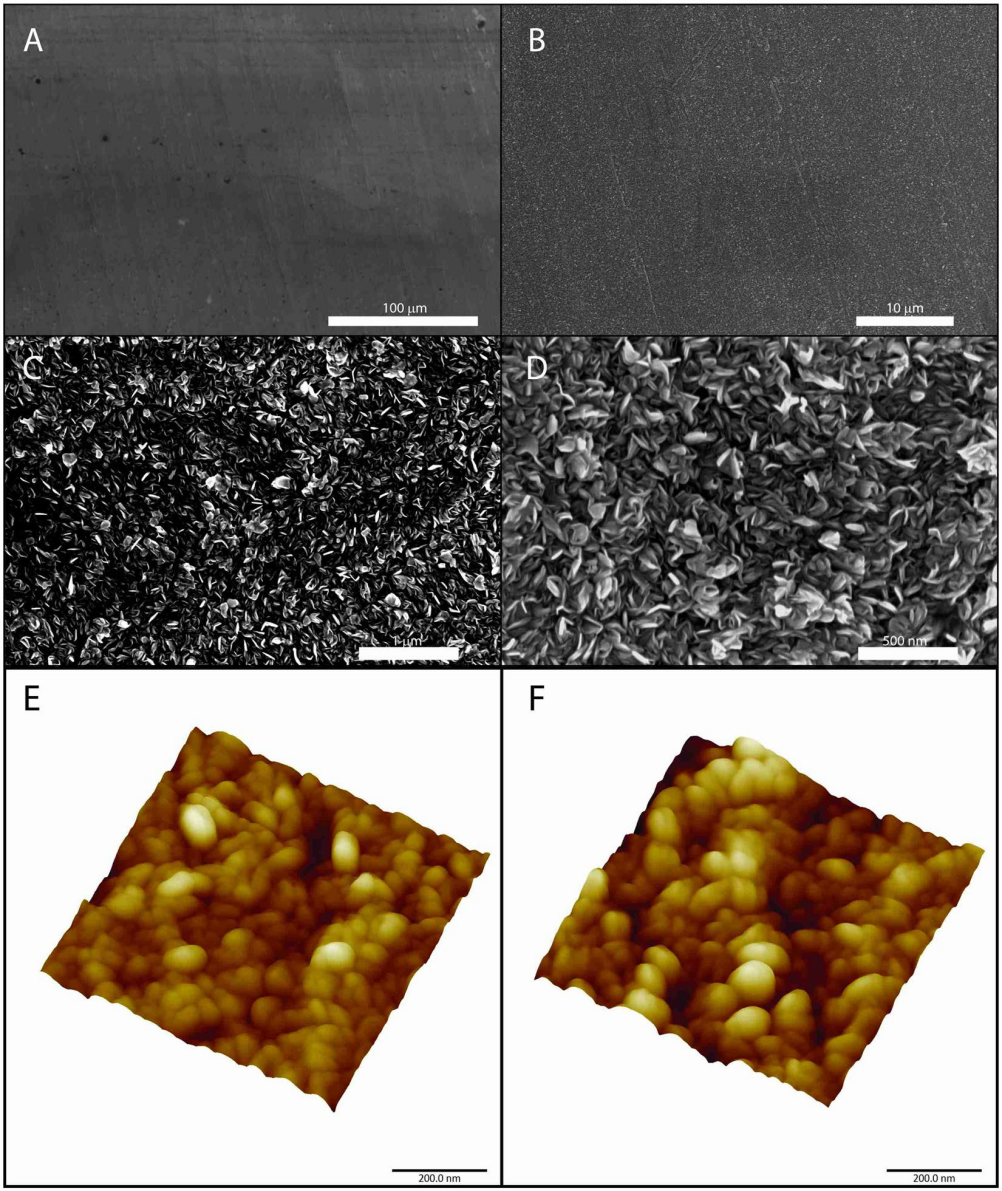
Scanning electron microscopy (SEM) (**A**–**D**) and atomic-force microscopy of VA-MoS_2_ #1 (**E**,**F**).

The high quality of the film is confirmed by SEM (Fig. 4A–D) on the whole sample. The roughness surface morphology of VA-MoS_2_ #1 is measured by atomic force microscopy (AFM) (Fig. 4E,F). The pristine Mo foil was polished before synthesis. After synthesis, the surface displays randomly oriented vertical flakes, which is consistent with the polycrystalline nature of the film. The roughness reaches the mean value of 21.4 nm.

The crystalline orientation of the film should impact the lithium intercalation. MoS_2_ flakes with micron-scale thickness (comparable to the CVD-grown samples) display a planar orientation (PO) while laminated on roughened copper foil used as a current collector for the Li-ion battery (LIB). MoS_2_ flakes on Cu were imaged on a scanning electronic microscope (Fig. S3). The “in-plane” images show the homogeneous layer of MoS_2_ flakes (Fig. S3A and B), whereas the cross-section shows the PO of the flakes (Fig. S3C and D). The film is formed from a single layer of flakes 500 nm thick with a good adhesion to the current collector. The orientation of the film is confirmed by Raman spectroscopy which displays the typical in-plane Mo−S phonon mode (E^1^_2g_) and the out-of-plane Mo-S phonon mode (A^1^_g_) (Fig. S4). The A_1g_ to E_g_ ratio is close to 1:2 ratio for the PO-MoS_2_^25^ while the VA-MoS_2_ displays a 1:3 ratio revealing the edge terminated nature of the film, i.e. the vertical alignment^28^. The same MoS_2_ flakes were also formulated in a slurry with a binder and carbon additive. The addition of a polymer and carbon black yields a thicker film with random orientation of the flakes.

**Figure 5:**
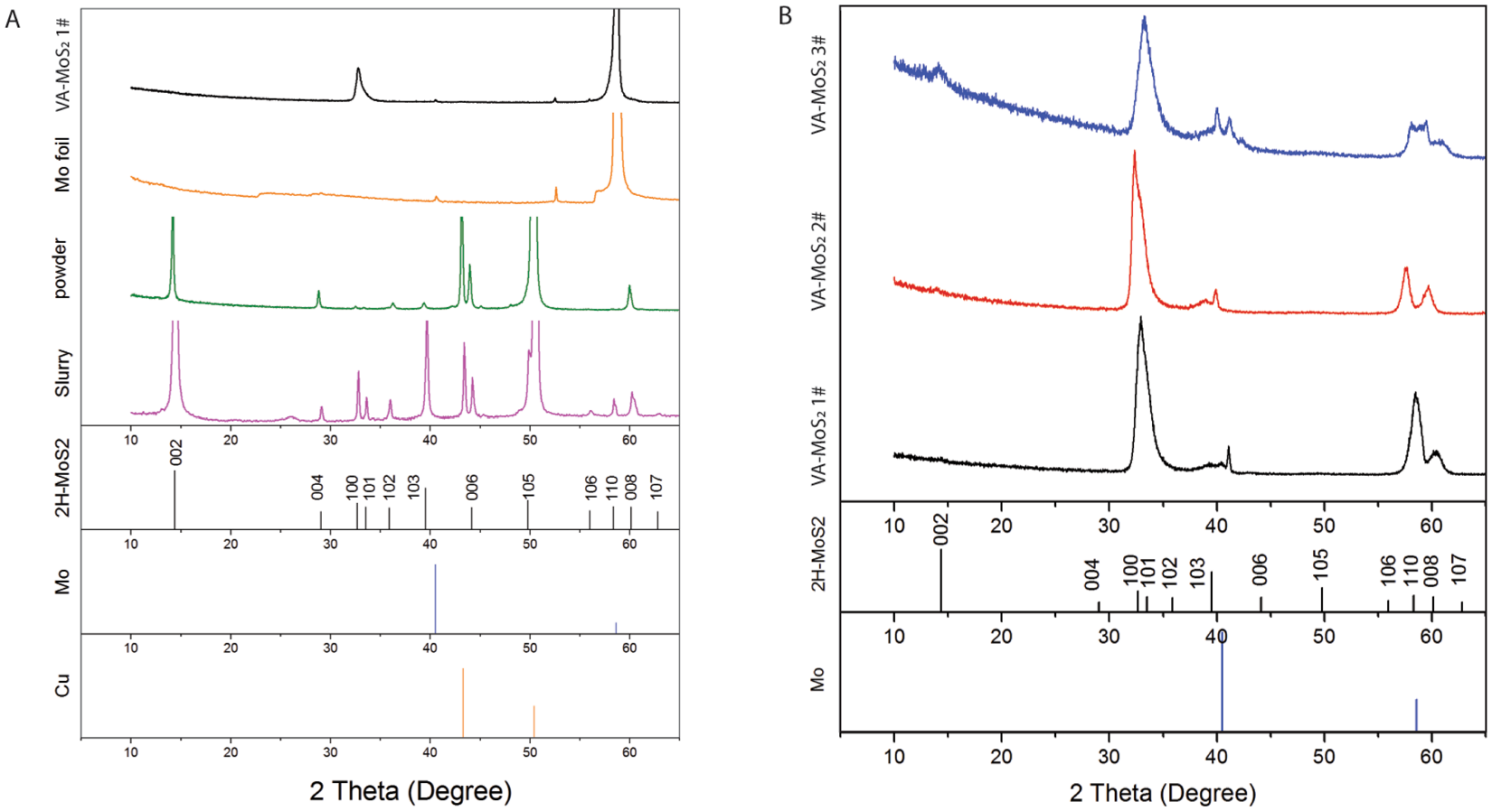
X-Ray diffraction (XRD) patterns of MoS_2_ films in slurry on Cu, PO-MoS_2_ on Cu and VA-MoS_2_ #1 on Mo (**A**); Grazing incidence-XRD (GIXRD) of CVD-grown samples (**B**).

X-ray diffraction (XRD) was carried out on VA-MoS_2_, PO-MoS_2_ and randomly oriented MoS_2_ flakes in the slurry (Fig. 5). Bragg-Brentano (θ–2θ) XRD of the MoS_2_ slurry exclusively shows the reflections assigned to the 2H-MoS_2_ phase (ICCD #00-037-1492) together with the reflections of the copper foil underneath (Fig. 5A). In the case of PO-MoS_2_ flakes on Cu foil, the XRD only displays the reflections from the basal planes (002), a clear sign of the preferential orientation of the flakes parallel to the Cu foil. In contrast, the CVD-grown sample lacks the (002) reflection and shows two main reflections: (100) of the 2H-MoS_2_ and (200) of the Mo foil. The XRD pattern confirms the orientation growth observed in HRTEM with vertically aligned basal planes and a growth along the (100) direction. Grazing incidence XRD (GIXRD) on the CVD-grown samples allows focusing on the MoS_2_ film itself, without the Mo background (Fig. 5B).

### Electrochemical characterization

**Figure 6:**
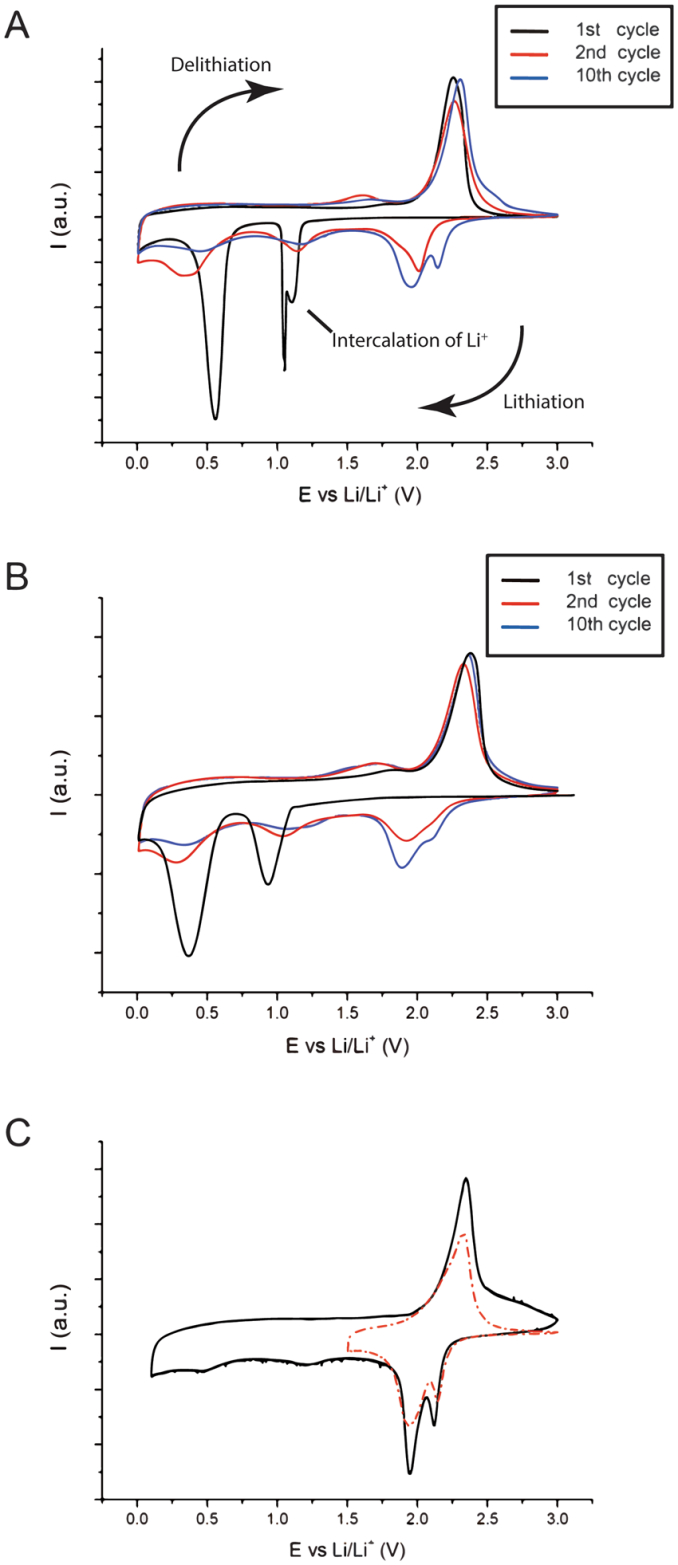
Cyclic voltammograms (1, 2 and 10) at 0.1 mV/s versus Li metal of VA-MoS_2_ #1 (**A**) and PO-MoS_2_ (**B**); cyclic voltammograms (50) of VA-MoS_2_ #1 in wide (black line) and narrow (red line) potential window (**C**).

All the electrodes were tested for Li storage under similar conditions with MoS_2_ as the working electrode and lithium as the counter-electrode and reference electrodes in a coin cell with a glassy paper separator impregnated with an EC/DMC 1 M LiPF_6_ electrolyte (see Experimental Section). Figure 6 displays the cyclic voltammograms (CV) over the range of 0.01–3.0 V versus Li/Li^+^. During the first cycle, VA-MoS_2_ #1 shows lithiation at 1.1 and 0.55 V and delithiation at 2.25 V (Fig. 6A). In the following cycles, the low potential peaks fade and the main lithiation-delithiation occurs between 2.2 and 1.8 V. This behavior points to the formation of a surface-electrolyte interphase (SEI) during the first cycle, followed by reversible intercalation processes in the following cycles. MoS_2_ flakes show similar behavior with slightly lower values for the lithiation potentials and higher values for the delithiation ones at the same scanning rate, revealing more sluggish kinetics (Fig. 6B). On the VA-MoS_2_ #1 sample, the potential was narrowed to 1.5 to 3.0 V after a few stabilizing cycles and the same faradic peaks as in the wide potential window are observed (Fig. 6C). Interestingly, the faradic peaks correspond to the electrochemistry of sulfur and not the intercalation of Li in MoS_2_. Therefore, most of the faradic capacity in the material can be obtained on a narrower potential window.

**Figure 7:**
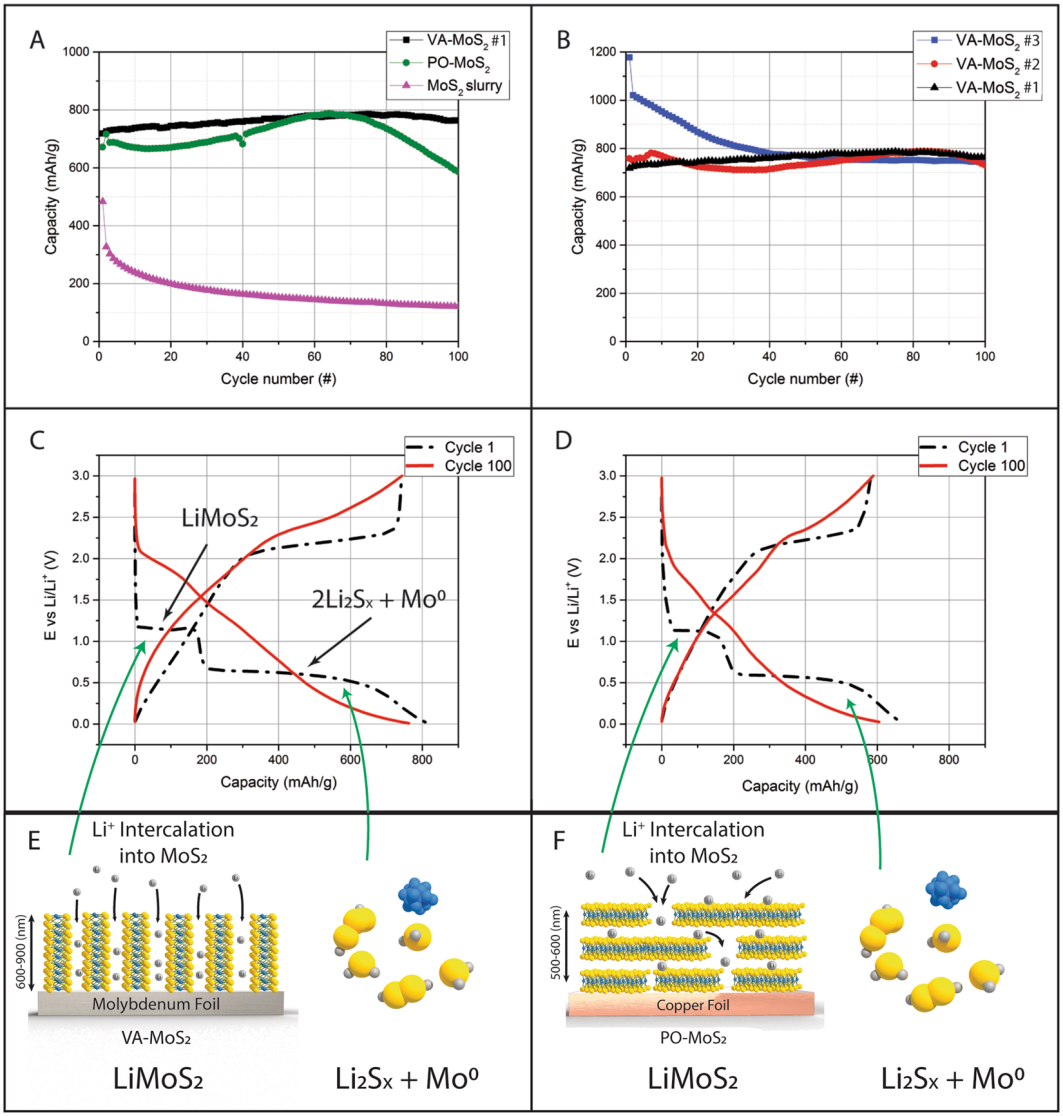
Specific capacity from galvanostatic measurements at a C/3 rate of VA-MoS_2_ #1, PO-MoS_2_ and slurry electrodes (**A**); Specific capacity at a C/3 rate of VA-MoS_2_ #1 (black triangle), VA-MoS_2_ #2 (red dots) and VA-MoS_2_ #3 (blue squares); voltage profile of cycle 1 (black dotted line) and cycle 100 (red plain line) for VA-MoS_2_ #1 (**C**) and PO-MoS_2_ (**D**); lithiation mechanism in VA-MoS_2_ (**E**) and PO-MoS_2_ (**F**).

The galvanostatic measurements on the samples are measured at a C/3 rate (3 hours charge and discharge) for at least 100 cycles for all samples (Fig. 7). The specific capacity of VA-MoS_2_, PO-MoS_2_ and MoS_2_ slurry is compared. Binder-free MoS_2_ (VA and PO) perform far better than the slurry, whose capacity rapidly fades from an initial 500 mAh/g value (Fig. 7A). Binder-free MoS_2_ displays a stable specific capacity of more than 700 mAh/g with a tendency to fade after 65 cycles for the PO-MoS_2_. The VA-MoS_2_ does not show any fading after 100 cycles and its specific capacity reaches 750 mAh/g. The different CVD-grown samples display almost the same specific capacity within a statistical error bar (Fig. 7B). The only observable discrepancy (a higher initial capacity for VA-MoS_2_ #3) arises from different conditioning of the cell which has not gone through CV measurements. These high specific capacities cannot be explained by a simple intercalation mechanism since it would involve only one lithium atom per MoS_2_ unit (~200 mAh/g). A specific capacity of 670 mAh/g corresponds to four lithium atoms. The potential of the vertically aligned sample displays two plateaus at 1.2 and 0.6 V for the first discharge (Fig. 7C, black curve). The first lithiation matches the intercalation of one Li atom in MoS_2_ and the second plateau corresponds to the insertion of three Li atoms. However, the second insertion does not correspond to an intercalation process but probably to conversion towards the Mo and Li_2_S phases. After 100 cycles, the specific capacity is retained but the voltage profile is smoother. Similar behavior is observed for PO-MoS_2_, which indicates that the insertion mechanism does not vary with the crystallographic orientation.

**Figure 8:**
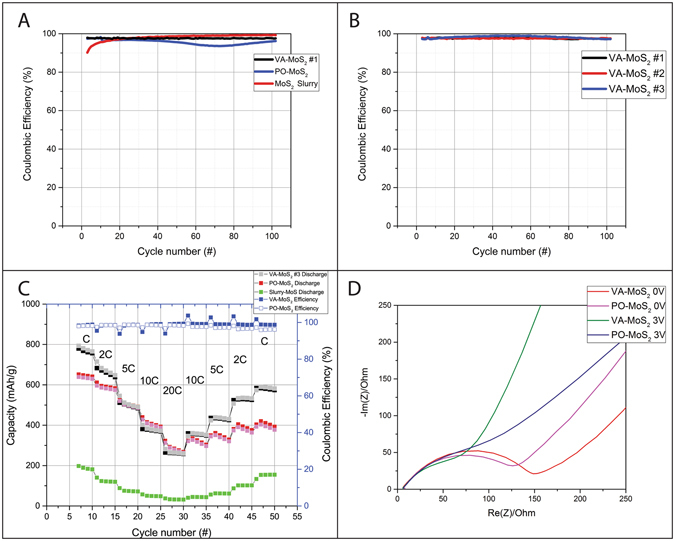
Coulombic efficiency at C rate of VA-MoS_2_ #1 (black), PO-MoS_2_ (red) and slurry (blue) (**A**); Coulombic efficiency at C rate of VA-MoS_2_ #1 (black), VA-MoS_2_ #2 (red) and VA-MoS_2_ #3 (blue) (**B**); C-rate of VA-MoS_2_ #1 (black), PO-MoS_2_ (red) and slurry (green) with the corresponding coulombic efficiency for VA-MoS_2_ #1 (blue); Nyquist plots of the VA-MoS_2_ #1 and PO-MoS_2_ after 10 cycles at 0 V and 3 V vs Li metal (**D**).

Figure 8 displays the Coulombic efficiency for each of the LIBs. The VA sample reaches values of 98% Coulombic efficiency, much higher than the PO flakes (94–96%) (Fig. 8A). The values are low compared to the acceptable standards in LIBs since the electrodes consist of thin films. Nevertheless, the comparison between the Coulombic efficiencies shows the benefits of the CVD growth regardless of film thickness (Fig. 8B). Figure 8C reveals the kinetics of the lithium insertion during a C-rate experiment. The current density gradually increases and then decreases for a corresponding charge duration of 1 hour and, finally, 3 min. It is interesting to note that the insertion kinetics for the CVD-grown samples is not improved, despite the vertical alignment which favors the Li intercalation between the basal planes.

To further demonstrate this point, electrochemical impedance spectroscopy (EIS) has been carried out on the batteries during 30 cycles. The typical Nyquist plots of the VA-MoS_2_ #1 and PO-MoS_2_ are shown in Fig. 8D after 10 cycles, at 3 V. For each EIS spectrum, two regions can be clearly seen: the semi-circle region corresponds to the charge transfer resistance at high frequency; the linear region (Warburg) corresponds to the Li-ion diffusion into the active material. The VA and PO electrodes shows similar charge-transfer resistance, indicating fast and highly efficient faradaic reactions for the two electrodes. The vertical alignment shows a slight enhancement of the Li-ion diffusion in the Warburg region with a higher slope compared to the planar orientation.

**Figure 9:**
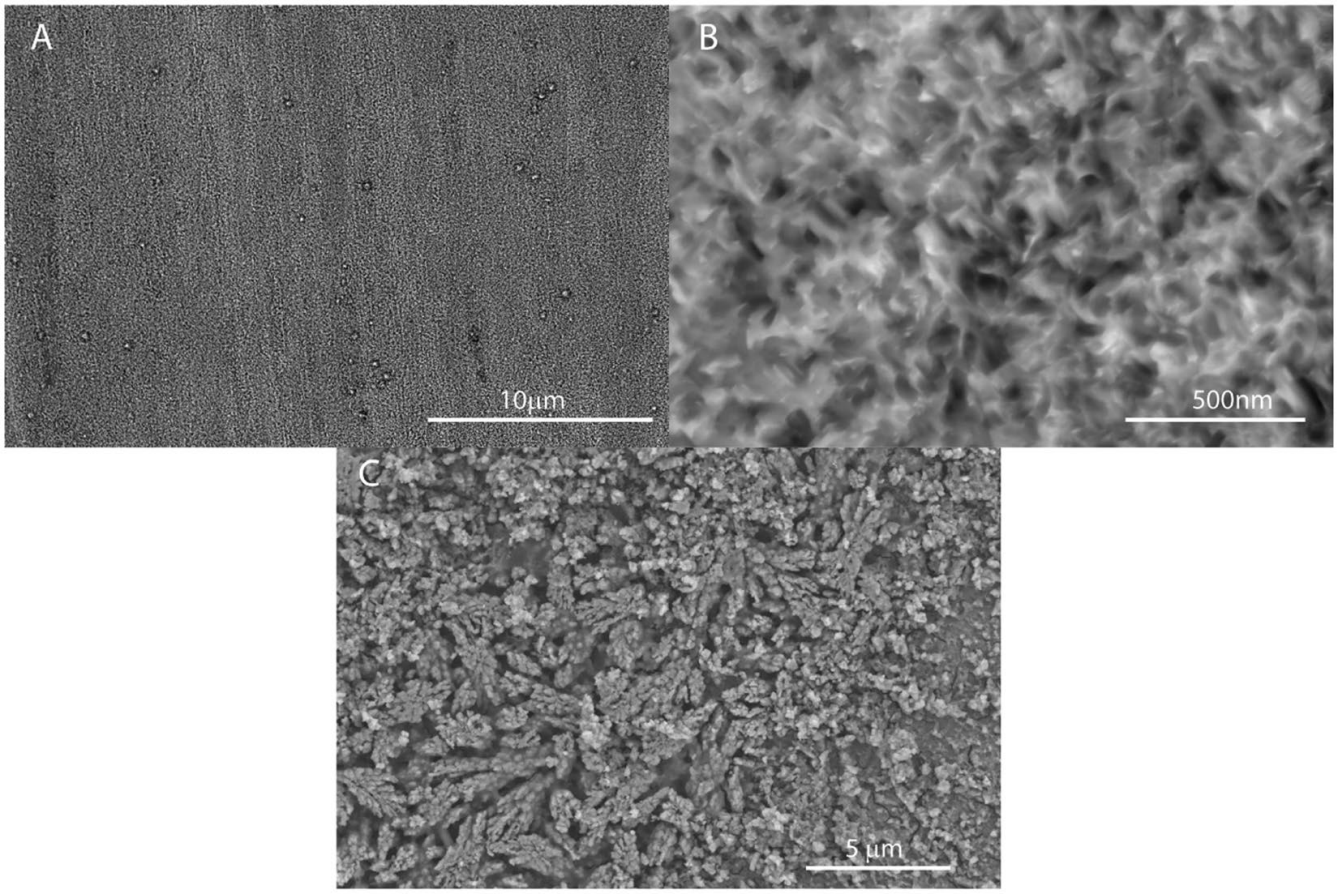
Scanning electron microscopy (SEM) of VA-MoS_2_ #1 after 10 cycles.

Furthermore, the post-mortem SEM analysis of the electrodes demonstrate that the vertical alignment remains even after multiple cycles (Fig. 9). The flakes of VA- MoS_2_ #1 after 10 cycles do have a preferred orientation even if the lithiation delithiation processes induce some disorder and a loose packing. A similar behavior has been observed on the PO-MoS2 which display a planar orientation of the flakes after 10 cycles as shown in supporting information (Fig. S6).

## Discussion

CVD growth of vertically aligned VA-MoS_2_ has been previously demonstrated on very thin samples. In the present study, the process is applied to much thicker films and VA 2H-MoS_2_ is grown with a thickness ranging between 500 and 1,000 nm. The layers are perfectly aligned and grow along the (100) orientation from the Mo foil, which itself has a preferential (200) orientation. However, no direct correlation can be drawn from the lattice parameters and we cannot claim epitaxial growth.

The electrochemistry of LTMDs has recently been extensively studied in exfoliated systems. In most of the publications, the authors correlate the high specific capacities to the low dimensionality of the materials. Nevertheless, metal sulfides, whether in the form of layered materials or not, have been studied in the bulk form and show a two-step lithiation mechanism. Goodenough *et al*. brought evidence from galvanostatic cycling and crystallographic data in favor of an intercalation process followed by the conversion of the materials towards metal and Li_2_S^48^. *This conversion mechanism is clearly verified quantitatively in our study where the specific capacity reaches the theoretical limit of 800 mAh/g*, *with a 1 to 3 ratio between the two lithium insertions*, *corresponding to the formation of LiMoS*_*2*_ *by intercalation and Mo* + *Li*_*2*_ *S by conversion* (Fig. 7E,F).

The high specific capacity obtained in a wide potential window (0–3 V is not of practical use as an electrode material) is retained in a narrower window (1.5–3 V), with most of the capacity at about 2 V. The conversion reaction turns out to be an alloying mechanism involving sulfur as the electroactive material. Elemental sulfur is the most promising element for future development of high-energy cathodes. Recent research papers have shown that the combination of LTMDs, namely, MoS_2_ with elemental sulfur, displays excellent stability even after hundreds of cycles^49^. The role of Mo has yet to be fully understood but the outcomes of our findings underline a generic concept for any LTMD. The demonstration of the full theoretical specific capacity in VA-MoS_2_ paves the way to high energy devices based on VA-LTMDs.

In comparison, the thin PO-MoS_2_ films also show a high specific capacity reaching the same theoretical value, but the specific capacity cannot be sustained more than 70 cycles. The main benefits arising from vertical alignment are the higher stability in cycling and Coulombic efficiency. Since the samples display similar thickness, the orientation must be responsible for the discrepancies. All the VA samples show better performance than the PO ones although the differences are not as extensive as one could have predicted. This assessment is obvious from analyzing the first electrochemical process, the intercalation of Li between the basal planes of MoS_2_ being very similar for the two orientations (Fig. 7C,D). In the conversion process that follows, the relationship between the structure and the conversion reaction is far more elusive and would require in-depth operando experiments to probe the electrode structure during cycling. Our preliminary results from EIS and post-morten SEM show that the cycling tends to conserve the film orientation. The VA alignment benefits to the Li ion diffusion and explains the differences observed between the two different orientations. These results are consistent with other materials grown as nano-walls and used as electrodes for LIBs.

In summary, CVD-grown VA-MoS_2_ films show a high reversible capacity of 800 mAh/g versus lithium, which corresponds to the theoretical limit of MoS_2_. The specific capacity is stable for more than 100 cycles at C/3 rate and reveals the full conversion to Mo and Li_2_S. The CVD process is developed to grow 2H-MoS_2_ films hundreds of nanometers thick with vertically aligned (VA) stacking directly from the Mo foil. The film shows large domains of perfectly aligned basal planes of 2H-MoS_2_ orthogonal to the foil. Noteworthy, both binder free MoS_2_ electrodes, planary oriented or vertically aligned, show high reversible capacity and the advantages of the vertical alignment become obvious while comparing with randomly oriented MoS_2_ electrodes with binder. Comparing the binder free electrodes, the VA electrodes show better capacity retention than their PO counterparts, which fail after 70 cycles and the vertical alignment allows faster charge-discharge rates while maintaining a high specific capacity (C-rate measurements). Apart from their interesting electrochemical properties, the CVD of dense and thick VA-LTMD films pave the way towards high performance energy storage devices.

## Methods

### VA-MoS_2_ synthesis

Molybdenum foil (0.05 mm thick, 99.95%, STREM) was polished and carefully washed. The polishing of the foil to a surface roughness of 21 nm was achieved with 50-nm alumina (see Supplementary Information), followed by cleaning with acetone, iso-propanol and 2 M HCl. The Mo pieces were introduced into a one-inch quartz tube. Sulfur (Sigma-Aldrich CAS #7704-34-9) was introduced into a boat in a different heat zone. The furnace was cleared of gas until a base pressure of Torr was reached, and then purged with nitrogen at 100 sccm for 10 minutes. Mo was then heated to 500 °C and S was heated to 145 °C. A temperature of 750 °C was maintained for 24, 48 or 72 hours and then the sulfur was cooled down to ambient temperature. The Mo foil was then cooled for over 2 hours.

### Electrode preparation

VA-MoS_2_ was used without further treatment by cutting disks from the foil. MoS_2_ powder (Sigma-Aldrich, <2 μm, 99%) was laminated onto a roughened copper foil (Oxygenfree, SE-Cu58, Schlenk Metallfolien GmbH & Co. KG) to yield PO-MoS_2_. The powder was brushed evenly over the surface (binder-free electrode: PO-MoS2). The slurry was made with 70% MoS_2_ powder (Sigma-Aldrich, <2 μm, 99%), 10% carbon black and 20% polyvinylidene fluoride (PVDF) from N-methyl pyrrolidone (NMP). The slurry was stirred overnight and then spread with a hand coater (wire rod diameter of 0.05 mm) on the Cu foil.

### Characterization

A focused ion beam (FIB) (FEI, Helios 600) was used to cut the CVD grown samples into lamellas for transmission electron microscopy (TEM) observation. High-resolution TEM (HRTEM) images were obtained by a JEOL JEM-2100 (LaB6) operated at 200 kV. Atomic force microscopy (AFM) measurements were carried out using a Bio FastScan scanning probe microscope (Bruker AXS). All images were obtained using the tapping mode with a FastScan-A (Bruker) silicon probe (spring constant of 18 N/m). For image processing and surface roughness analysis, the Nanoscope Analysis Software was used. Rutherford backscattering spectroscopy (RBS) was performed using a 1.7 M V Pelletron accelerator from NEC. RBS and particle-induced x-ray emission (PIXE) spectra were acquired simultaneously. The PIXE data were analyzed using the GUPIX software. Raman spectroscopy was carried out on Horiba LabRam HR evolution with a 532 nm Laser.

X-ray diffraction (XRD) images were collected on a Rigaku Smartlab XRD in Bragg-Brentano (θ–2θ) and grazing-incidence XRD (GIXRD) mode. For the GIXRD, grazing-incidence parallel beam optics (PB/PSA) with a fixed incident angle of 0.8° was used. The X-ray generator was operated at 40 kV and 30 mA with Cu-Kα radiation (λ = 1.54 Å). Scanning electron microscopy (SEM) images were collected on a FEI Quanta FEG250 on the pristine electrodes and after washing with dry dimethyl carbonate and drying on the cycles electrodes.

### Electrochemistry

The electrodes were tested in coin-type cells (2523, NRC, Canada) vs. lithium metal (Chemetall Foote Corporation, USA) using glassy paper (Hollingsworth & Vose, BGM03010). The electrolyte solution was ethylene carbonate/dimethyl carbonate (EC/DMC) (1/1 ratio) with 1 M LiPF6 (purelyte, Ube industries). The cells were assembled in an argon-filled glovebox with a purifying system (MBraun GmbH, Germany) (Fig. S5).

Cyclic voltammetry was performed with a Bio-Logic VMP3 multi-channel potentiostat at 0.1 mV/s between 0.01–3.0 V and 1.5–3.0 V vs. Li^+^/Li with EIS. The coin-cells were cycled at 30 °C using an Arbin MSTAT BT2000 battery cycler at a C/3 rate, and the C rate was tested under the same conditions after 100 cycles.

## Electronic supplementary material

Supplementary information
