# Supplementary material for: On the impact of Vertical Alignment of MoS2 for Efficient Lithium Storage
Source: Sci Rep. 2017 Jun 12;7:3280. doi: 10.1038/s41598-017-03453-x (PMC5468230; doi:10.1038/s41598-017-03453-x)
Supplement: Supplementary file 1 — Supplementary information [file 41598_2017_3453_MOESM1_ESM.pdf]

Additional information

On the impact of Vertical Alignment of MoS_2 for Efficient Lithium Storage

Victor Shokhen[1], Yana Miroshnikov[1], Gregory Gershinsky[1], Noam Gotlib[2], Chen Stern[2], Doron Naveh[2,*], David Zitoun[1,*]

[1] Department of chemistry and [2] Faculty of Engineering
Bar Ilan Institute of Nanotechnology and Advanced Materials (BINA), Bar Ilan University, Ramat Gan, 52900, Israel

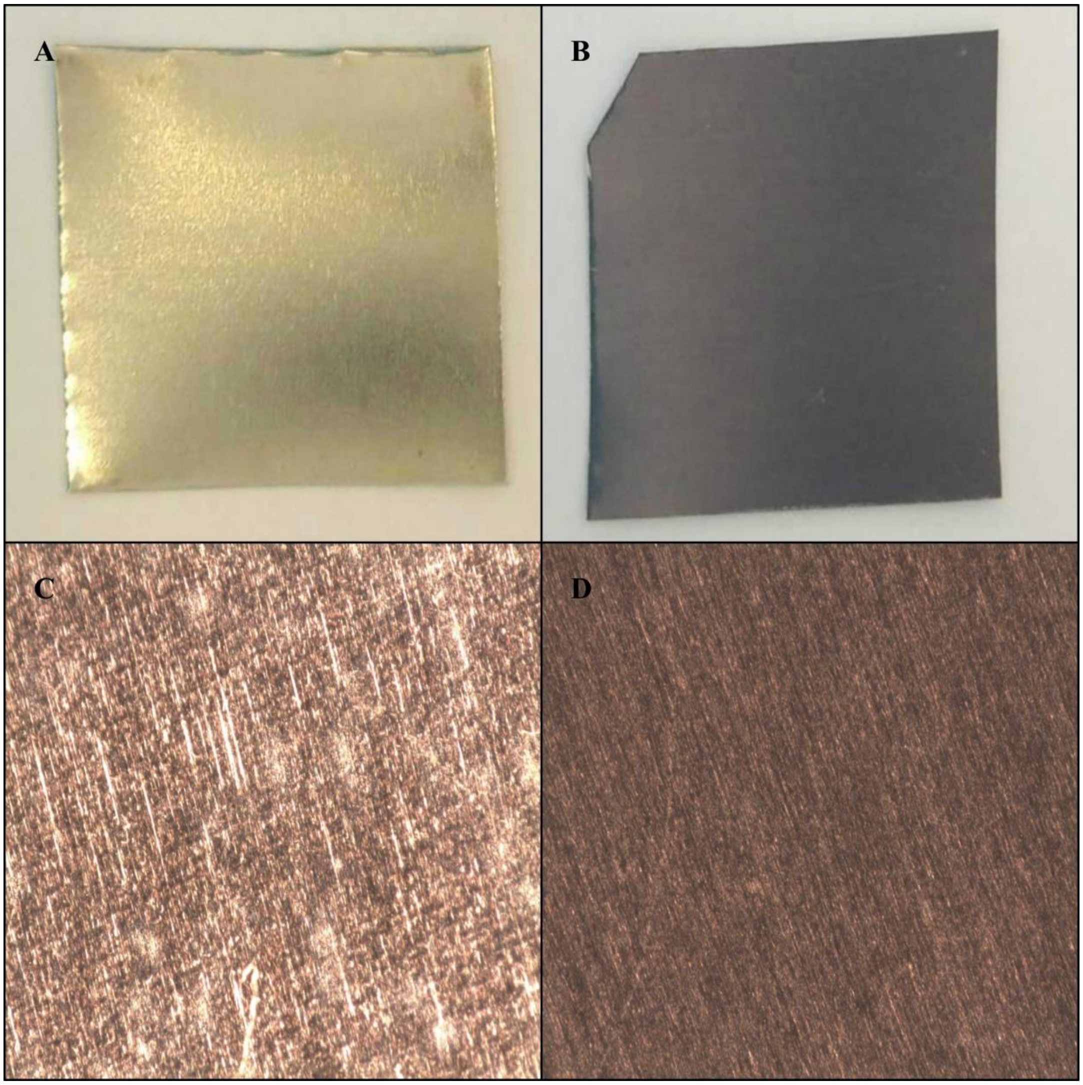

Figure S1. Photographs of the pristine Mo foil 10*10 mm^2 (A & C) and the VA-MoS_2 grown by CVD (B & D)

# of the sample	S *10^{18} at/cm^2	Mo , *10^{18} at/cm^2	Thickness, nm	Model: Mo S_y / Mo
**VA-MoS_2 2#**	2.418	1.45	801.5	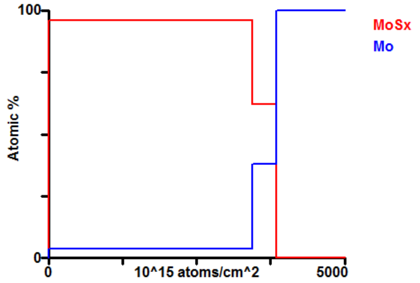
**VA-MoS_2 3#**	1.568	0.9796	526.5	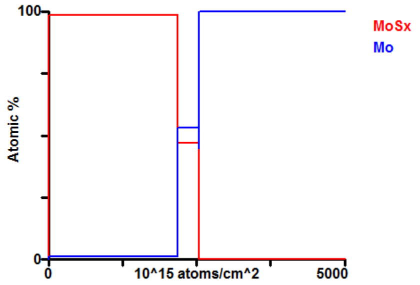

Figure S2. Particle-induced x-ray emission (PIXE) analysis of the VA-MoS_2 grown by CVD

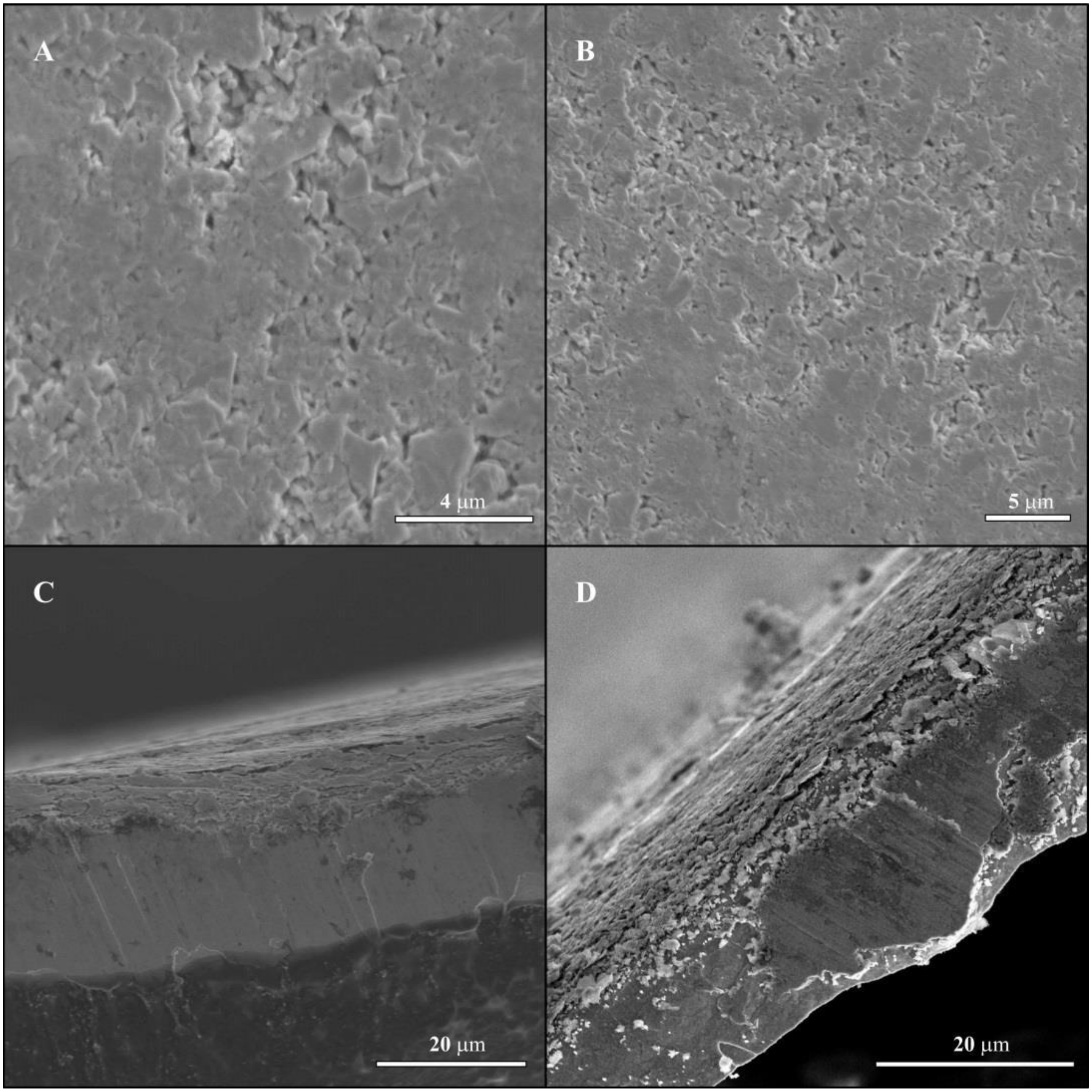

Figure S3. SEM of the PO-MoS_2 electrodes on Cu, “in plane” (A-B) and cross-section (C-D)

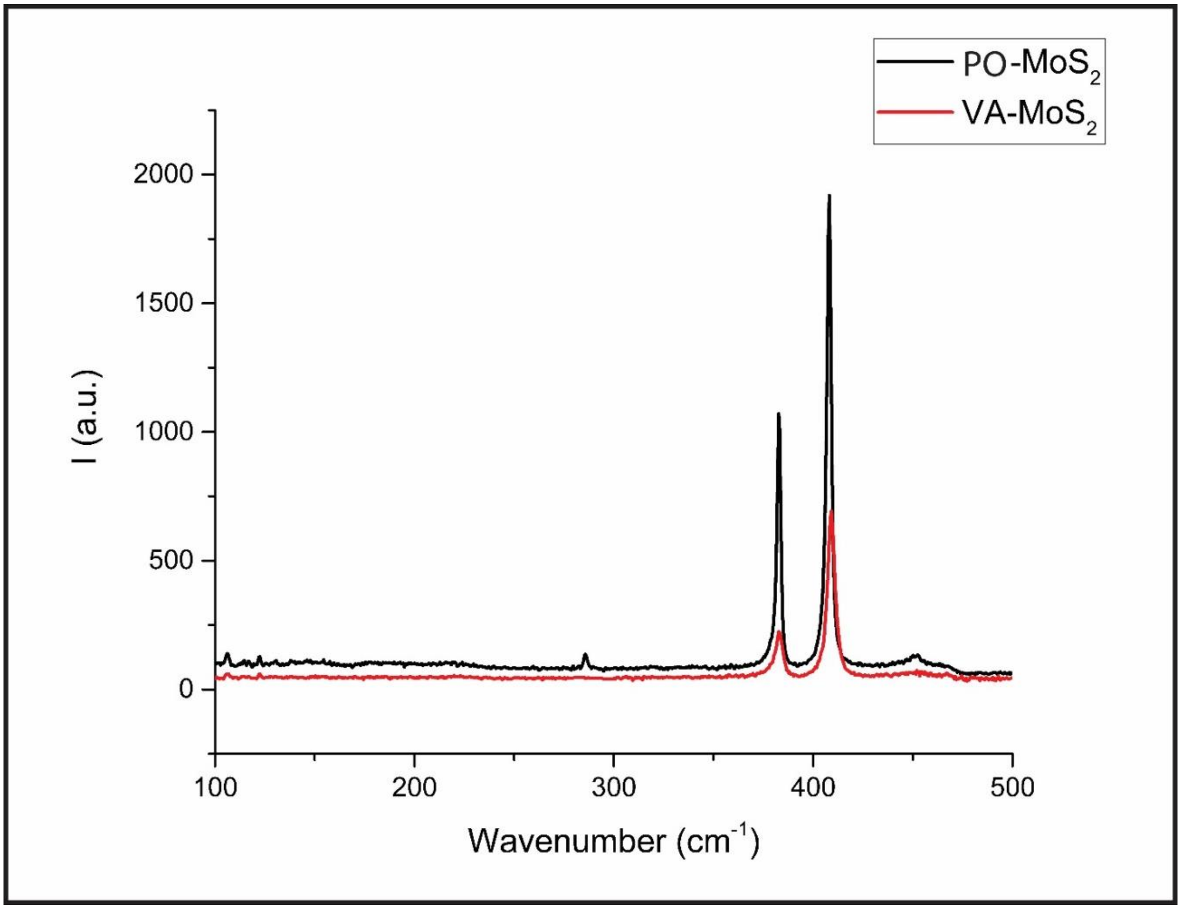

Figure S4. Raman spectra of the PO-MoS_2 and VA-MoS_2

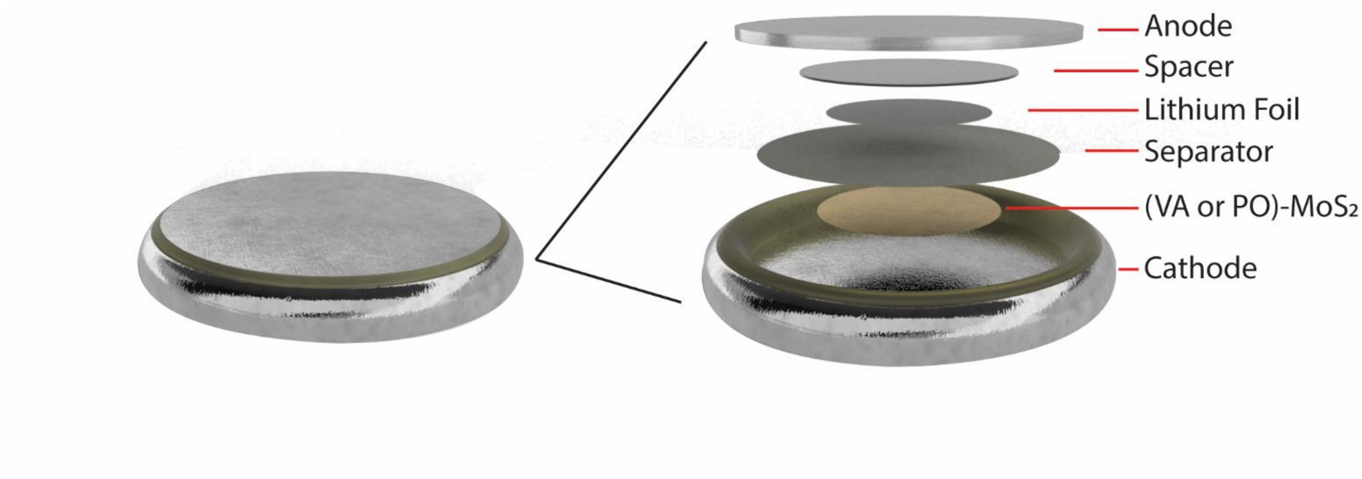

Figure S5: Coin cell assembly

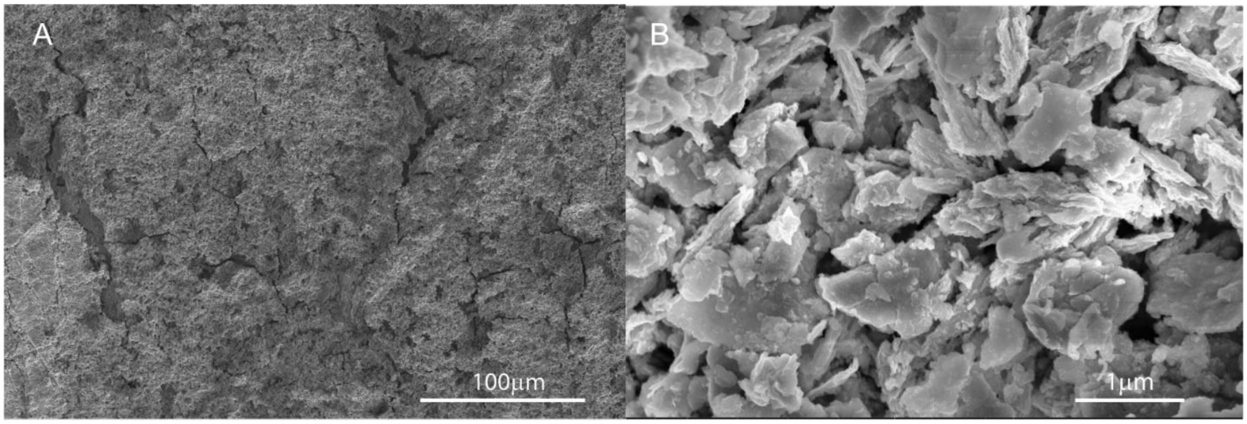

FigureS6. Scanning electron microscopy (SEM) of cycled PO-MoS_2
